# Significance and Characteristics of* Listeria monocytogenes* in Poultry Products

**DOI:** 10.1155/2019/7835253

**Published:** 2019-04-18

**Authors:** Abdollah Jamshidi, Tayebeh Zeinali

**Affiliations:** ^1^Department of Food Hygiene and Aquaculture, Faculty of Veterinary Medicine, Ferdowsi University of Mashhad, Mashhad, Iran; ^2^Social Determinants of Health Research Center, Faculty of Health, Birjand University of Medical Sciences, Birjand, Iran

## Abstract

*Listeria monocytogenes* is one of the most common foodborne pathogens. Poultry meat and products are of the main vehicles of pathogenic strains of* L. monocytogenes* for human. Poultry products are part of the regular diet of people and, due to nutrient content, more content of protein, and less content of fat, gain more attention. In comparison with red meat, poultry meat is more economical. So, it had a greater rate of consumption especially in barbecue form in which the growth of bacterium is favored. Subtyping of* L. monocytogenes* isolates is essential for epidemiological investigation and for identification of the source of contamination. In the following review, the main facet of presence of* L. monocytogenes* in poultry will be discussed. Most pathogenic serotypes of* L. monocytogenes* were detected in different products of poultry meat. Unfortunately, these isolated pathogens had sometimes resistance to commonly used antibiotics which were used for treatment of human infection.

## 1. Characteristics of* Listeria monocytogenes*

*Listeria* spp. are small gram-positive rod (0.5–4 *μ*m in diameter and 0.5–2 *μ*m in length), non-spore-forming, facultative anaerobic, catalase-positive, and oxidase-negative organisms.* Listeria* has tumbling motility at 20–25°C due to peritrichous flagella. Based on somatic (O) and flagellar (H) antigens, 13 serotypes were identified in* Listeria monocytogenes* (*L. monocytogenes*) including 1/2a, 1/2b, 1/2c, 3a, 3b, 3c, 4a, 4ab, 4b, 4c, 4d, 4e, and 7 [1]. With the aid of multiplex PCR assay, four major serovars of* L. monocytogenes* strains can be categorized into four distinct serogroups, IIa (serovars 1/2a, 1/2c, 3a, and 3c), IIb (1/2b, 3b, 4b,4d, and 4e), IIc (1/2c and 3c), and IVb (4b, 4d, and 4e) by targeting four marker genes [2]. Food or food production environment is commonly contaminated with serotypes 1/2a, 1/2b, 1/2c, and 4b. The optimum growth temperature of* L. monocytogenes* is 30–37°C, but it can survive between 0 and 45°C.* L. monocytogenes* can multiply at refrigerator temperatures, is resistant to disinfectants, and adheres to various surfaces [1]. Once introduced into the processing plants, it is able to survive and remain for a long period under adverse conditions [1]. In the food industry,* L. monocytogenes* is able to form biofilm which can act as a potential source of contamination [3].* L. monocytogenes* is a widely distributed organism in nature, with main reservoirs of soil and forage. Moreover, it was isolated from healthy humans and animals or infected domestic and wild animals [4].

## 2. Listeriosis

*L. monocytogenes* is the main cause of foodborne listeriosis in humans. Rarely, foodborne infections were reported by* L. ivanovii* and* L. seeligeri*. Strains of* L. monocytogenes* have different pathogenic potential, as some strains are very virulent, whereas some of them are noninfectious agents [4, 5]. Determination of the pathogenic potential of* L. monocytogenes* is important from food safety and public health perspective [6]. Identification of virulent strains can be achieved through tracing some genes directly related to pathogenicity of* L. monocytogenes* [7].* L. monocytogenes* enters into host cells by use of a family of surface proteins called internalins, especially InlA and inlB. Moreover, InlC and InlJ also participate in the postintestinal stages of* L. monocytogenes* infection [8]. Putative internalins of* L. monocytogenes* are encoded by inlC (lmo1786) and inlJ (lmo2821) genes. The etiologic organism of human's listeriosis harbors inlJ (lmo2821) [9].* L. monocytogenes* carries a pore-forming toxin named listeriolysin O (LLO) (a 58 KDa protein-encoded by hlyA gene) which is vital for virulence of the bacterium [4]. LLO lyses the membrane of the vacuole and finally assists the entrance of* L. monocytogenes* into the cytoplasm [4]. Several methods had been used to assess the virulence of* L. monocytogenes*. Some of them include mouse virulence assay, cell culture, and use of specific genes and proteins [8].  Table 1 shows some specific genes used to determine the virulence of* L. monocytogenes* isolates in poultry.

Foodborne listeriosis has three main clinical features, namely, meningitis, septicemia, and abortion. In healthy humans it can cause febrile gastroenteritis, but in susceptible persons (children, elderly, immune-compromised and pregnant women) it may lead to septicemia and meningitis [1].

Listeriosis is the fourth commonly zoonotic disease in Europe, with the annual incidence of 0.41 cases per 100,000 population [10]. In Asian countries, reports of listeriosis rarely exist due to the failure of detection or report. Also, it may be due to lower incidence rate or exclusion of listeriosis for differential diagnosis by clinicians. However,* L. monocytogenes* has been regarded as one of the etiological factors of spontaneous abortions and stillbirth in India [11].

People more than 65 years old and neonates had the highest rates of infection with* L. monocytogenes* [12]. Maternal transmission to newborns was reported in 79% of cases. Listeriosis has the highest case fatality rate among foodborne diseases [10]. Isolation of* L. monocytogenes* from different kinds of RTE foods made it a remarkable foodborne pathogen [13, 14].

## 3. Subtyping of* L. monocytogenes*

Due to diverse strains of* L. monocytogenes*, subtyping of isolates for population genetics, source tracking, and the epidemiologic investigation is crucial for control and prevention of listeriosis. Typing of* L. monocytogenes* is needed to identify the sources of contamination and investigate foodborne listeriosis outbreaks [15, 16]. Phenotypic and genotypic subtyping are the two main methods which were used by researchers. As a phenotypic method, serotyping is generally used for* L. monocytogenes* strains related to disease outbreaks. Due to the involvement of only three serotypes in listeriosis outbreaks and low discriminatory power of serotyping in distinguishing of serotypes 4a, 4b, and 4c, serotyping does not have enough power for subtyping of* L. monocytogenes* [16]. So, PCR-based subtyping procedure such as Random Amplification of Polymorphic DNA-Polymerase Chain Reaction (RAPD-PCR), Repetitive Extragenic Palindromes-PCR (REP-PCR), Enterobacterial Repetitive Intergenic Consensus-PCR (ERIC-PCR), and Pulsed Field Gel Electrophoresis (PFGE) gain more attention these days. RAPD assay amplified some random region in the* L. monocytogenes* genomes which generate distinct patterns. RAPD is more cost effective and faster than other typing methods, especially for low number of strains. RAPD-PCR technique is one of the main methods for bacterial strain characterization [15–18]. Enterobacterial Repetitive Intergenic Consensus-PCR (ERIC-PCR) is a highly reliable, simple, and economic method which is able to produce clear fingerprint in* Listeria* [19]. ERIC-PCR analysis can separate the isolates of the same serotype. Also, it is capable of differentiating* L. monocytogenes* isolates which were detected in one sample with similar serotype [18].

Restriction Fragment Length Polymorphisms (RFLP) amplified one or some of the housekeeping or virulence-associated genes (e.g., hly, actA, and inlA) of* L. monocytogenes* and then digested PCR products with restriction enzymes [16]. It needs a low copy number of DNA to perform the experiment [16]. But, it has a lower discriminatory power and should be used along with other subtyping techniques and it is also more expensive than RAPD assay [20]. One of the other methods of genotyping of* L. monocytogenes* isolates is Amplified Fragment Length Polymorphisms (AFLP) method. In AFLP, digestion of DNA of isolates was done with two restriction enzymes including EcoRI, MseI, or TaqI [16]. One of the main advantages of AFLP is the high discriminatory power of this test [20]. In contrast, the pitfall of this method is low precision in fragment sizes, which leads to lower reproducibility [16].

PFGE is a tool in which, by exposing large DNA fragment to changing electric field, isolates were subtyped. This technique was more discriminatory than AFLP, but is more time consuming, expensive, and labor intensive in comparison with AFLP [20].

*L. monocytogenes* also has some randomly dispersed, repetitive sequence elements, such as repetitive extragenic palindromes (REPs) of 35–40 bp with an inverted repeat. These regions provide some useful points for strain distinction of* L. monocytogenes* isolates. Using REP-PCR, the origin of isolates was identified. It has an equal level of discrimination to PFGE. So it is suggested as a suitable technique for rapid typing of these isolates [16].

## 4. *Listeria* in the Poultry

### 4.1. Prevalence of* Listeria* Spp. and* L. monocytogenes* in the Poultry

One of the main vehicles of* Listeria* is poultry flocks which can spread the organism into the environment and poultry carcass due to unhygienic practice [21]. Occasionally,* Listeria* was isolated from the feces of poultry and chicken.* Listeria* spp. were detected in various poultry products [13, 22–25]. According to other studies, 8% to 99% of poultry products were contaminated with* Listeria* spp. [13, 24, 26, 27].

*L. monocytogenes* has been previously reported from different poultry products from raw products to cooked ones [13, 22, 28–35]. Schäfer et al. (2018) reported the contamination rate of breast and thigh samples of chicken as 8.64 and 44.19%, respectively [36]. 12.7 % of turkey meat was positive for* L. monocytogenes* [37].  Table 2 shows the contamination rate of poultry meat and products with* Listeria* spp. and* L. monocytogenes.*

According to Table 2, raw poultry meat and products were more contaminated with* L. monocytogenes* than cooked ones.

### 4.2. Serotypes of* Listeria monocytogenes* in Poultry

Serotypes 1/2b and 3b (serogroup IIb) of* L. monocytogenes* were the predominant isolated serotypes (52.77%) in chicken carcasses in Iran, and IVa serogroup which contains 4a and 4c serotypes also was detected in 27.77% of chicken carcasses [6]. The most common serotype in poultry products in the USA [38] was the same. But in another study, serotype 4b has been reported as the most common serotype in poultry products which was detected in 44.9% of the samples, while the prevalence of serotype 1/2b was 10.2% [33].

The prevalence of serogroup IVb was 2.77% and 12.5% in chicken carcasses [6] and RTE foods, respectively [14]. Human listeriosis is mainly caused by 1/2a, 1/2b, and 4b serovars of* L. monocytogenes*. However, 4b serotype was not commonly found in foods [6].

Fresh packed turkey meat samples were contaminated with* L. monocytogenes* serotypes as follows: 4b (or 4d, 4e) (51.4%), 1/2a (or 3a) (27.0%), and 1/2b (or 3b) (21.6%) [39]. However, serotype 4b was frequently isolated from turkey meat and legs, while 1/2b was prevalent in turkey breast samples [39].

About 16.66% of the chicken carcasses sampled in Iran were contaminated with serogroup IIa containing 1/2a, 3a, 1/2c, and 3c serotypes [6]. Another serological study on poultry products reported 1/2a serotype in 40.8% and 1/2c serotype in 4.08% of samples [33]. In other studies, 1/2a serotype was the predominant serotype in poultry products of Portugal and Estonia [40, 41], while in Finland 1/2c was the major one [42].The identified serogroups in RTE foods were 1/2a, 3a and 1/2c, 3c with the rate of 65.6% and 21.9%, respectively [14]. Based on the above studies, poultry meat is a potential source of pathogenic serotypes of* L. monocytogenes*.

### 4.3. Antimicrobial Susceptibility of* Listeria monocytogenes*

*Listeria* spp. are resistant to antimicrobial agents due to widespread mobile genetic elements and conjugative transposons [33]. Twelve out of 36* L. monocytogenes* isolates were sensitive to 11 tested antimicrobial agents [22]. None of the isolates had resistance to ampicillin and vancomycin [22]. Some researchers observed resistance to ampicillin in* L. monocytogenes* isolates, but all of their isolates were sensitive to vancomycin [33]. Zeinali et al. (2017) observed resistance to erythromycin in 52.77% of* L. monocytogenes* isolates but, in another study, it was reported in 15.2% of the isolates [33]. 8 out of 23 of* L. monocytogenes* isolates had resistance to erythromycin [37]. Resistance to penicillin is a common finding in a number of studies [22, 33, 37, 43]. Moreover, high susceptibility of* L. monocytogenes* to ampicillin and penicillin is also reported [22, 27, 44–46]. Tetracycline is an antimicrobial agent with frequent use in poultry farms and also the treatment of human's infection. Resistance to this agent is always observed in* L. monocytogenes* [13, 22, 33, 47, 48]. A low number of isolates were resistant to gentamycin [22, 49]. Standard therapy of listeriosis is done by use of ampicillin or penicillin G together with an aminoglycoside such as gentamicin. The second line of treatment belongs to trimethoprim. Resistance to trimethoprim in* L. monocytogenes* contributes to the pIP823 plasmid. There is a high susceptibility to this agent among* L. monocytogenes* isolates from foods [22, 49]. Most of the* L. monocytogenes* isolates had multidrug resistance. Fortunately, they are mostly sensitive to commonly used antibiotics which were used to cure human listeriosis.

### 4.4. Typing of* L. monocytogenes* Isolates in Poultry

Isolates of the* L. monocytogenes* with the same RAPD cluster belonged to different serogroup [15, 54–56]. Four different clusters were distinguished among 26 isolates of* L. monocytogenes* from chicken carcasses through RAPD analysis with three different primers, namely, OPM-01, HLWL 74, and D8635 [57]. These 26 isolates of* L. monocytogenes* had 16 antibiogram patterns [57].

*L. monocytogenes* isolates with similar pulse-types were classified in the same cluster in the RAPD assay. They were also clonally related [14]. Different laboratories used RAPD test for subtyping of* L*.* monocytogenes* isolates [14, 58], including isolates from different poultry processing plants [58, 59].

Several isolates of RTE foods were typed by RAPD, although they were indistinguishable by REP-PCR [14]. Twenty-eight isolates of* L. monocytogenes* from chicken meat had 27 RAPD types. They were resistant to three or more antimicrobial agents [60].

Fifteen isolates of* L. monocytogenes* from ducks had three antibiogram patterns, five RAPD clusters, and three singletons. So, RAPD had a higher power in distinguishing isolates [58].

Chicken and human isolates of* L. monocytogenes* were classified in five clusters in RAPD assay [54]. All human isolates were categorized in one cluster [54]. These isolates had different serogroup [54]. It was a common finding in other studies [15, 55, 56, 61]. It may be due to amplification of unspecific loci in RAPD test [15]. Most genetic similarities were seen among isolates which had common sampling area [54]. The same RAPD cluster was seen in some Lactobacillus strains from common source [62]. Discrimination power of RAPD test is higher than serotyping [54, 56]. Isolates in the same RAPD profile had different serotypes and were detected in different areas [15, 32, 54, 55, 63, 64].

29 isolates and 5 reference strains of* L. monocytogenes* were grouped into 4 clusters and 1 singleton by REP-PCR [65]. There was a high genetic diversity among isolates. According to Shi et al. (2015), isolates belonging to the same serotype and origin had the same cluster in REP-PCR. 15 isolates of* L. monocytogenes* from ducks and their environments were typed by RAPD and REP. They were categorized in 5 clusters and 3 singletons, and 2 clusters and 3 singletons, respectively. This finding proposed the suitability of these tools for discrimination of strains [58]. Soni et al. (2012) also observed that clinical isolates of* L. monocytogenes* had similar ERIC and REP fingerprints but are quite different from the water and milk isolates [47]. Oliveira et al. (2018) found 12 pulsotypes among 38 isolates of* L. monocytogenes* [17]. 40 isolates of* L. monocytogenes* produced 10 different fingerprint profiles in ERIC-PCR. Similar fingerprint were seen for isolates of the same sample, but there was two strains in one sample with different fingerprints [18].* L. monocytogenes* had a high genetic diversity, and for good differentiation of isolates the use of at least two subtyping approaches is necessary.

## 5. Conclusion

In conclusion, from the food safety perspective, the presence of* L. monocytogenes* in the poultry meat and products is a multifaceted potential hazard. This is due to, firstly, some barbecued and fried foods based on chicken meat which may lead to the survival of* L. monocytogenes* in final products and, secondly, the presence of multidrug resistance isolates which transfer the antibiotic resistance to community. Also, some of the isolates were pathogenic serotypes that play a major role in human listeriosis outbreaks. Subtyping data revealed the heterogeneous nature of the* L. monocytogenes* isolates. RAPD, REP-PCR, and ERIC-PCR have a considerable discriminatory power and are cost effective and less tedious and time consuming.

## Figures and Tables

**Table 1:** Specific genes used to determine the virulence of *L. monocytogenes.*

genes	Sample	reference
prfA	black-headed gull	[50]

hly	black-headed gull; chicken carcass	[6, 50]

actA/plcB	black-headed gull	[50]

inlA/inlB	black-headed gull	[50]

iap	black-headed gull	[50]

InlC	chicken carcass	[6]

inlJ	chicken carcass	[6]

**Table 2:** Prevalence of* Listeria *spp. and *L. monocytogenes *in poultry meat and products.

Type of Product	Number	Method of Analysis	Contamination Rate (%) of Listeria spp.	Contamination Rate (%) of *L. monocytogenes*	Region	year	Reference
fresh chicken carcasses	160	Culture/PCR	47.5%	9.37%	Jordan	2011	[13]

fresh chicken carcasses	200	Culture/PCR	40%	18%	Northeast of Iran	2017	[22]

Frozen Poultry	6	Culture/PCR	0%	0%	Center of Iran	2008	[23]

Fresh poultry	66	Culture/PCR	4.5%	0%	Center of Iran	2008	[23]

RTE Chicken product	120	Culture/PCR	54.17%	30%	Jordan	2011	[13]

Broiler wing meat	120	Culture/PCR	47.5%	45%	Turkey	2015	[25]

Raw poultry (chicken, Turkey, quail, ostrich, chicken liver)	199	Culture/PCR	34.7%	14.1%	Center of Iran	2012	[33]

Ready to cook (Barbecued chicken, Chicken schnitzel, Chicken nugget)	115	Culture/PCR	33%	12.2%	Center of Iran	2012	[33]

Ready to eat poultry product (Olivieh salad, Chicken sausage, Chicken burger)	88	Culture/PCR	30.7%	11.4%	Center of Iran	2012	[33]

raw poultry products	63	Culture/PCR	100%	41%	Portugal	2002	[51]

raw poultry products	772	culture	-	38.2%	Belgium	1999	[29]

Chicken carcasses	100	PCR	99%	38%	northern Greece	2011	[27]

Raw chicken	38	culture	-	34%	Sri Lanka	1995	[28]

Raw poultry products	15	culture	61.1%	22.2%	Nordic countries	2004	[52]

poultry minced meat	23	Culture/PCR	30.4%	4.35%	Poland	2005	[30]

raw chicken parts	70	Culture/PCR	51.4%	7.14%	Poland	2005	[30]

poultry meat heat-treated products	50	Culture/PCR	0%	0%	Poland	2005	[30]

Fresh and Frozen	99	Culture/PCR	-	19.2%	South Africa	2005	[31]

raw chicken	210	MPN/PCR	-	20%	Malaysia	2012	[34]

Fresh turkey meat	180	Culture/PCR	-	12.77%	Turkey	2011	[37]

frozen chicken meat	2327	Culture/PCR	-	2.5%	Thailand	2011	[53]

RTE chicken products	1273	Culture/PCR	-	0.2%	Thailand	2011	[53]

chicken offal (Liver, heart, gizzard)	216	MPN/PCR	-	26.39%	Malaysia	2013	[35]
